# Sustained Extracellular Electrical Stimulation Modulates the Permeability of Gap Junctions in *rd1* Mouse Retina with Photoreceptor Degeneration

**DOI:** 10.3390/ijms25031616

**Published:** 2024-01-28

**Authors:** Sophie Stürmer, Sylvia Bolz, Eberhart Zrenner, Marius Ueffing, Wadood Haq

**Affiliations:** Institute for Ophthalmic Research, University of Tuebingen, 72076 Tuebingen, Germany

**Keywords:** gap junctions, electrical synapse, neuronal networks, retinal degeneration, retinitis pigmentosa, electrical stimulation, retinal implants, artificial vision

## Abstract

Neurons build vast gap junction-coupled networks (GJ-nets) that are permeable to ions or small molecules, enabling lateral signaling. Herein, we investigate (1) the effect of blinding diseases on GJ-nets in mouse retinas and (2) the impact of electrical stimulation on GJ permeability. GJ permeability was traced in the acute retinal explants of blind retinal degeneration 1 (*rd1*) mice using the GJ tracer neurobiotin. The tracer was introduced via the edge cut method into the GJ-net, and its spread was visualized in histological preparations (fluorescent tagged) using microscopy. Sustained stimulation was applied to modulate GJ permeability using a single large electrode. Our findings are: (1) The blind *rd1* retinas displayed extensive intercellular coupling via open GJs. Three GJ-nets were identified: horizontal, amacrine, and ganglion cell networks. (2) Sustained stimulation significantly diminished the tracer spread through the GJs in all the cell layers, as occurs with pharmaceutical inhibition with carbenoxolone. We concluded that the GJ-nets of *rd1* retinas remain coupled and functional after blinding disease and that their permeability is regulatable by sustained stimulation. These findings are essential for understanding molecular signaling in diseases over coupled networks and therapeutic approaches using electrical implants, such as eliciting visual sensations or suppressing cortical seizures.

## 1. Introduction

The functional disorder of neuronal membrane channels leads to the impairment of sensory systems, such as the visual system, significantly impacting lifestyle. Hence, different strategies have been investigated to aid the channelopathies of the visual system; these strategies include gene editing (e.g., achromatopsia [1]), using drugs to gain control over the channel’s function (e.g., the photoreceptor’s cGMP channels [2]), and introducing functional channels into the cell membrane (e.g., channel rhodopsin [3,4]). Furthermore, advanced clinically approved applications for humans include electronic implants that excite neurons electrically (e.g., retinal [5,6] and cortical implants [7]) and photoreceptor cell transplantation (e.g., in the case of age-related macular degeneration [8]).

The success of these strategies is dependent on thorough knowledge of the functional state of the remnant diseased retinal network. Thus, the transmitter-mediated vertical signal transduction pathway in the blind retina has been investigated in several studies [9,10,11]. However, little is known about the lateral gap junction (GJ)-mediated communication pathway in the photoreceptor-degenerated retina [12,13]. GJs, also known as electrical synapses, facilitate direct intercellular connections via connexins (Cx), which allow the exchange of small molecules or ions (electrical signals). In a healthy retina, GJ-coupled cell networks (GJ-nets) are established mainly among cells of the same type, namely rod and cone photoreceptors (rPhR-cPhR net (Cx36)) and horizontal (HC-HC net (Cx57 and Cx50)), amacrine (AC-AC net (Cx36 and Cx45)), or ganglion cells (GC-GC net (Cx36)) [13,14,15]. GJs play an essential role in shaping the visual signal in a healthy retina, such as through control of the receptive field, adjustment to ambient light conditions, the rod signal-to-cone pathway, correlated firing of GC, global object and motion recognition, and regulation of circadian rhythm [12,16].

Overall, clear proof of the remaining functionality of GJ-nets in the photoreceptor-degenerated retina is lacking to date. While the anatomical data suggest altered GJ coupling due to neuronal sprouting [17,18,19], the functional studies indicate some remaining functionality [20,21,22,23].

Thus, this study aimed to investigate the GJ-nets in photoreceptor-degenerated *rd1* mouse retinas, a commonly used model in vision research to reflect the human blinding disease retinitis pigmentosa [17,24]. Thus, three crucial research questions were investigated: (1) For overall therapeutic approaches, are GJs present in the *rd1* retinas, and do they form functional GJ-nets? (2) Can the GJs’ permeability be modulated? (3) Specifically for the application of electrical implants, does sustained stimulation modulate GJ permeability and thereby influence the span of GJ-nets? Accordingly, we determined the GJ permeability in acute retinal explants by cut loading and diffusion of the tracer neurobiotin (adapted from Choi et al. [25]), which can permeate through GJ but *not* the cell membrane. Fluorescently tagging the tracer allowed the analysis of the tracer spread in histological immunofluorescent preparations. In the investigation of the effect of electrical stimulation on GJ permeability, sustained subretinal stimulation (with a single large electrode) was applied to acute *rd1* retinal explants in long-term experiments. Additionally, as a counter experiment, the GJs were blocked by the pharmaceutical agent carbenoxolone (CBX) [26,27].

Our approach unequivocally proved that *rd1* mouse retinas with photoreceptor degeneration facilitate functional GJ-nets of HC-HC, AC-AC, and GC-GC. Moreover, the conducted experiments are the first to demonstrate the modulatory effect of sustained subretinal stimulation on the permeability of different types of GJs in the retina.

Overall, these findings are of great relevance for therapeutic approaches in neuronal tissue facilitating GJs that are capable of the lateral spread of signals via ions (electrical charge) or small molecules (messengers). This study implies the necessity of rethinking, particularly when designing stimulation paradigms for electrical implants. The electrical stimulation-dependent modulation of GJ permeability may considerably alter the spatial and temporal characteristics of the perceived visual sensation or, in general, modulate the signaling properties of the excitable cells that form GJ-nets.

## 2. Results

### 2.1. GJ-Nets of Degenerated rd1 Mouse Retinas

**Figure 1:**
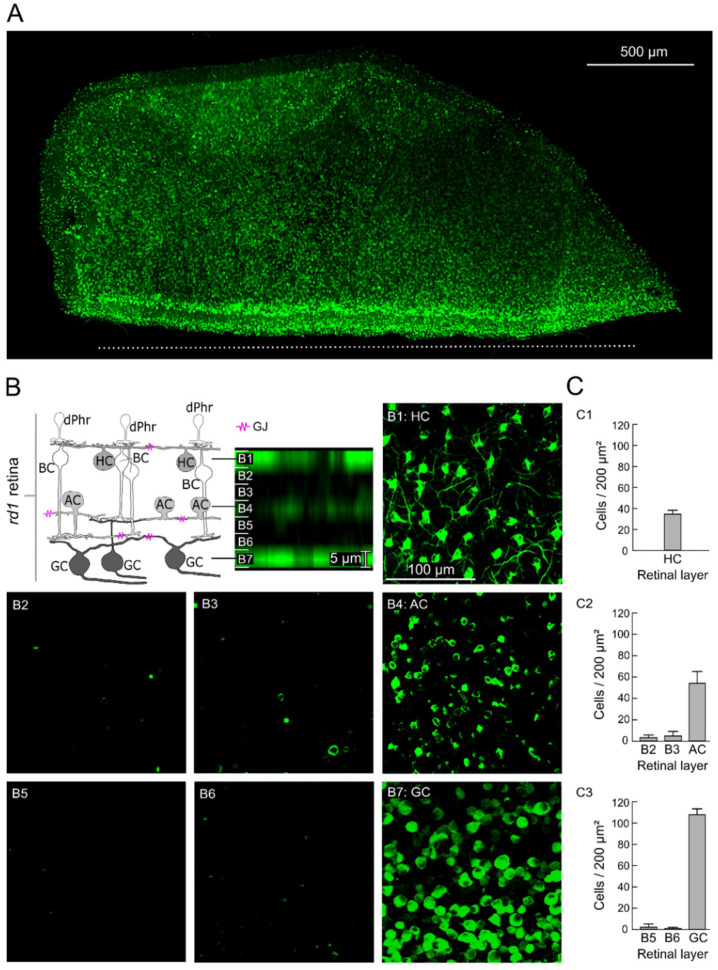
Visualization of the retinal gap-junction coupled cell networks using the tracer loading method. (**A**) Tracer-loaded *rd1* mouse retina. Representative histological fluorescence microscopy image of an *rd1* retinal explant (focal plane: outer retina) presenting cells loaded with the tracer neurobiotin, which can permeate gap junctions (GJs) *only*. The white dashed line indicates the razor-cut site and the retinal entry for the neurobiotin. (**B**) GJ-coupled cell networks (GJ-nets). Cross-section of a tracer-loaded retina (right, from (**A**), stack step 5 µm) in association with a retinal sketch (left). The seven stack focal planes (**B1**–**B7**) are three different GJ-nets (magenta in sketch) identified as horizontal (HC-HC net, **B1**), amacrine (AC-AC net, **B4**), or ganglion cell (GC-GC net, **B7**) GJ-net, and two zones between HC layer and AC layer (**B2**,**B3**) and between AC layer and GC layer (**B5**,**B6**). The scale bar in (**B1**) (100 µm) applies to (**B1**–**B7**). (**C**) Cell density. The number of cells per 200 µm^2^ in retinal layers (**C1**: HC (**B1**), **C2**: intermediate layers (**B2** and **B3**) and AC (**B4**), **C3**: intermediate layers (**B5** and **B6**) and GC (**B7**)), respectively. Error bars indicate ± standard deviation (*n* = 9 retinas). Abbreviations of retinal cells: dPhr: degenerated light-insensitive photoreceptor; BC: bipolar cell.

To assess the GJ-nets in the photoreceptor-degenerated *rd1* mouse retinas, we established the method for network tracing. The retinal explants were cut with a razor blade to open an entry for tracer diffusion and were incubated in a solution of the GJ-net tracer neurobiotin. As a result, in the histological fluorescence microscopy images, a pronounced labeling of the retinal tissue was observed (Figure 1A). As the tracer neurobiotin can permeate through GJ *only* (not the cell membrane [26,27,28]), the diffusion of the tracer from the cut site throughout the periphery of the untreated retinal tissue is interpreted as GJ-net mediated that are connected by open GJs (Figure 1A; measured diffusion depth: 1.41 ± 0.20 mm; *n* = 9 retinas).

The retinal GJ-nets (Figure 1B) of the horizontal (HC-HC net; Figure 1(B1)), amacrine (AC-AC net; Figure 1(B4)), and ganglion cells (GC-GC net; Figure 1(B7)) were identified and discriminated using the retina-topographic characteristics of the labeled cells, such as position in the distinctive layer, cell shape, and cell size (Figure 1B). Through imaging of the outer retina, the tracer-labeled cell network was identified as an HC-HC net (Figure 1(B1)). The HCs formed a characteristic regular mosaic of large cells, with dendrites forming a widely coupled cell network (Figure 1(C1); 34 ± 3.91 HCs/200 µm^2^; *n* = 9 retinas). The GC-GC net (Figure 1(B7)) was identified by imaging the GC layer (opposite to the outer retina), with characteristic GC axons and different GC shapes (Figure 1(C3); 108 ± 4.59 GCs/200 µm^2^; *n* = 9 retinas). In addition, we found labeled cells between the HC and GC layers (Figure 1(B4)). We identified these as ACs (AC-AC net) due to their relatively equal distance from the GC layer (15 µm; Figure 1(B5,B6)) and HC layer (15 µm; Figure 1(B2,B3)). The relatively cell-free zone (between the GC layer and AC layer (Figure 1(B5,B6)): 1.15 ± 2.07 cells and between the HC layer and AC layer (Figure 1(B2,B3)): 3.67 ± 3.07 cells) and the existence of *no* cell overlap with the other layers made it evident that this was a different cell layer. The AC-AC net was not as pronounced as the other two nets; nevertheless, it was identified in all the experiments as a unique intermediate cell layer (Figure 1(C2); 54 ± 11.69 ACs/200 µm^2^; *n* = 9 retinas).

### 2.2. GJs of Degenerated rd1 Mouse Retinas Are Functional

**Figure 2:**
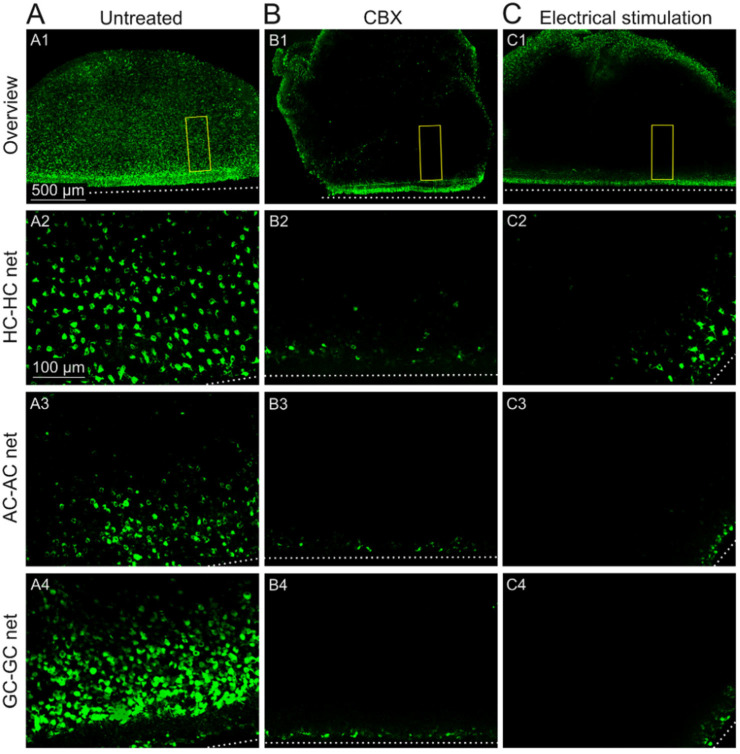
Tracer diffusion through retinal networks after GJ block or electrical treatment. (**A**) Retinal GJ-net, with tracer neurobiotin loaded in histological fluorescence images of untreated retina (as established in Figure 1): (**A1**) overview (focal plane: outer retina), (**A2**) HC-HC net, (**A3**) AC-AC net, and (**A4**) GC-GC net. (**B**) Pharmaceutical block of the GJ-net. Image sequence (**B1**–**B4**) of retinal layers (as in (**A**)) treated with the GJ inhibitor carbenoxolone (CBX). (**C**) Electrical modulation of the GJ-net. The image sequence (**C1**–**C4**) of retinal layers (as in (**A**)) treated with electrical stimulation (1 V for 2 h). The scale bar in (**A1**) (500 µm) applies to images in the first row; the scale bar in (**A2**) (100 µm) applies to all other images. Yellow squares in overviews (**A1**,**B1**,**C1**) mark the region of interest (ROI) for the estimation of the TDR (Figure 3) of the retinal razor-cut site (white dashed line), with the entry of the GJ tracer toward the inner retina.

To investigate the functionality of the GJ-nets in the *rd1* mouse retinas, we modulated their permeability using the GJ inhibitor CBX. Bath application of CBX for 1 h inhibited tracer diffusion from the cells at the cut site into the GJ-coupled cells, abolishing the GJ-nets and providing proof of functional HC-HC and GC-GC nets in the *rd1* retina (Figure 2(A1–A4,B1–B4)).

**Figure 3:**
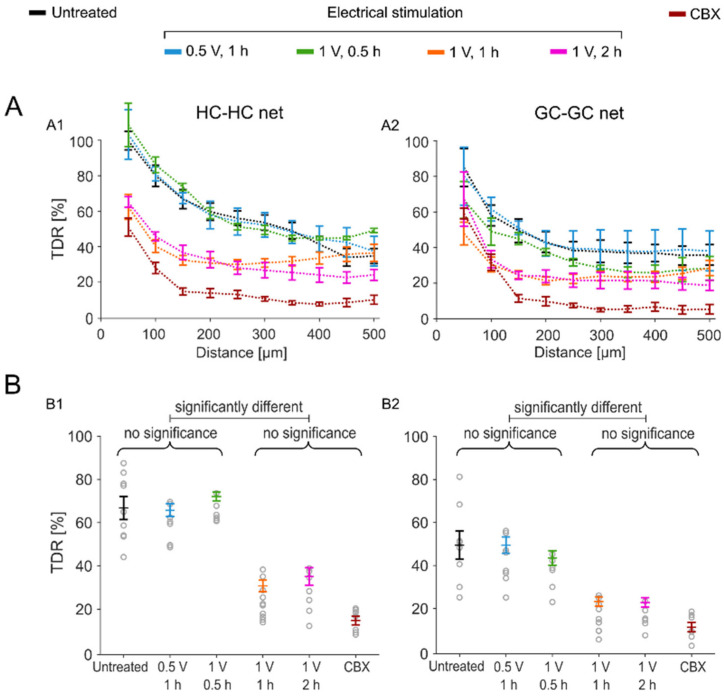
Effects of GJ blocker and electrical stimulation on retinal GJ permeability. (**A**) Spatial diffusion of GJ tracer. Assessment of TDRs (yellow ROIs in Figure 2) through the retinal GJ-net of (**A1**) HCs (HC-HC net) and (**A2**) GCs (GC-GC net). Three treatment conditions were tested with parameter variation: untreated, sustained stimulation (0.5 V for 1 h, 1 V for 0.5 h, 1 V for 1 h, or 1 V for 2 h), or bath application of pharmaceutical GJ inhibitor CBX (1 h). The X-axis represents the distance from the razor-cut site (see Figure 2; 50 µm steps). Retina numbers for each condition: untreated: *n* = 8; CBX: *n* = 7; and electrical stimulation: *n* = 31 (0.5 V for 1 h: *n* = 8; 1 V for 0.5 h: *n* = 6; 1 V for 1 h: *n* = 10; 1 V for 2 h: *n* = 7). (**B**) Impact of different treatments. Statistical evaluation of TDRs (from **A**) at the 150 µm mark for (**B1**) HC-HC net and (**B2**) GC-GC net. Error bars indicate the mean ± standard error of the mean (SEM). One-way analysis of variance (ANOVA) was applied to estimate the statistical significance, followed by Dunnett’s test for multiple comparisons (*p*-values are provided in Table S1).

**Figure 4:**
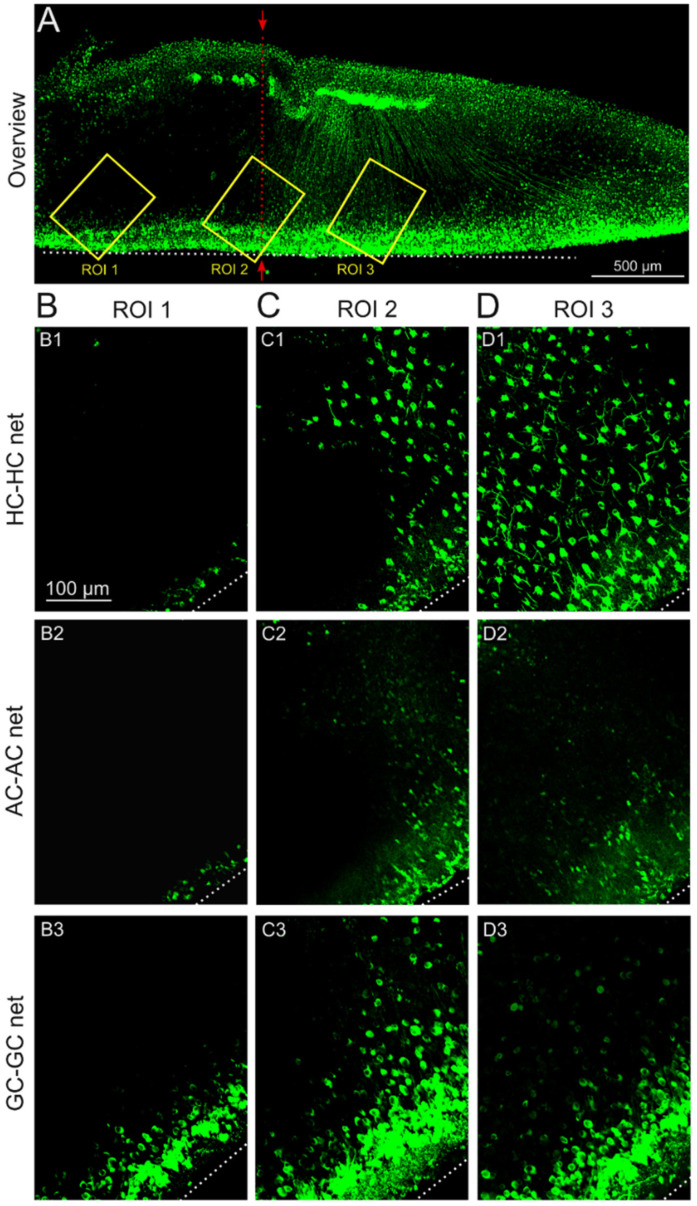
Stimulation electrode location-dependent retinal tracer diffusion. (**A**) Co-localized retinal stimulation. Histological fluorescence imaging of the GJ tracer neurobiotin throughout *rd1* retinal explants in the electrode overlap experiment (see methods), half stimulated and half untreated, marked by red arrows (1 Hz and 1 V stimulation for 1 h). Retinal GJ-nets were assessed at three ROIs (yellow squares) with respect to the electrode covering the retina: (**B**) covered area (ROI 1: HC-HC net (**B1**), AC-AC net (**B2**), and GC-GC net (**B3**)), (**C**) intermediate area (ROI 2 (cell layers as in (**B**))), and (**D**) uncovered area (ROI 3 (cell layers as in (**B**))). A white dashed line indicates the retinal razor cut for intrusion of the GJ tracer. The scale bar in (**B1**) (100 µm) applies to the image series of (**B**–**D**).

We assessed the most effective impact of the GJ blocker at a distance of 150 µm from the cut site, with a substantial drop in tracer diffusion rate (TDR) by approximately 87% (Figure 3A and Figure 4B; outer retina: 85%; GC layer: 89%; no more significant changes onwards from 150 µm). In contrast, at the same mark of 150 µm, the untreated condition showed only a decline of 42% in TDR (outer retina: 33%; GC layer: 51%). Tracer loading of the cells under 150 µm was not considered, as this was not *only* GJ-wise transmitted, although a clear effect in the decay of diffusion was detected (50 µm; HC layer: untreated = 99.74 ± 5.17% and CBX = 51.04 ± 5.04%; GC layer: untreated = 84.99 ± 10.73% and CBX = 59.15 ± 3.01%). It is conceivable that the observed cell loading could also be derived from the cells directly impaired by the cut (large cellular dendritic field).

### 2.3. Sustained Electrical Stimulation Affects the GJ Permeability of Degenerated rd1 Mouse Retinas

Through electrical stimulation treatment experiments, we investigated the impact of sustained stimulation on GJ permeability in *rd1* retinal explants. Subretinal stimulation was applied at various voltages for different durations (stimulation frequency: 1 Hz) using a single large electrode (diameter: 5 mm), which resembled an integrated large multielectrode array (see Section 4.3 Experimental Conditions). First, the application of balanced biphasic cathodic ± 1 V stimulation for 2 h diminished the tracer spread through the GJs in all the cell layers (Figure 2(C1–C4)). In the vicinity of the cut (at the 150 µm mark; see Section 3.2), no HC-HC or GC-GC nets were detectable, as was the case with the pharmaceutical inhibition experiments using CBX (Figure 3 and Table S1; TDR drop 2 h stimulation vs. untreated: outer retina: 77% (*** *p* < 0.001) and GC layer: 85% (*** *p* < 0.001)).

Shortening the stimulation duration from 2 h to 1 h still resulted in an effective reduction in GJ permeability that was similar to that of the 2 h condition (Figure 3; TDR drop of reduced 1 h stimulation vs. untreated: outer retina: 76% (*** *p* < 0.001) and GC layer: 84% (*** *p* < 0.001)). However, when the stimulation duration was reduced to 30 min, no significant effect of the stimulation on the GJ-nets, compared to the untreated GJ-nets, was observed at the 150 µm mark (TDR of reduced 30 min stimulation vs. untreated: outer retina: 2% (n.s.) and GC layer: 13% (n.s.)). Reducing only the stimulation strength to 0.5 V also did not affect the permeability of the GJ-nets in the *rd1* retinas (Figure 3; TDR of reduced 0.5 V stimulation vs. untreated: outer retina: 8% (n.s.) and GC layer: 7% (n.s.)).

### 2.4. Impact of the Stimulation-Induced Reduction in GJ Permeability Is Local to the Stimulation Electrode Position

**Figure 5:**
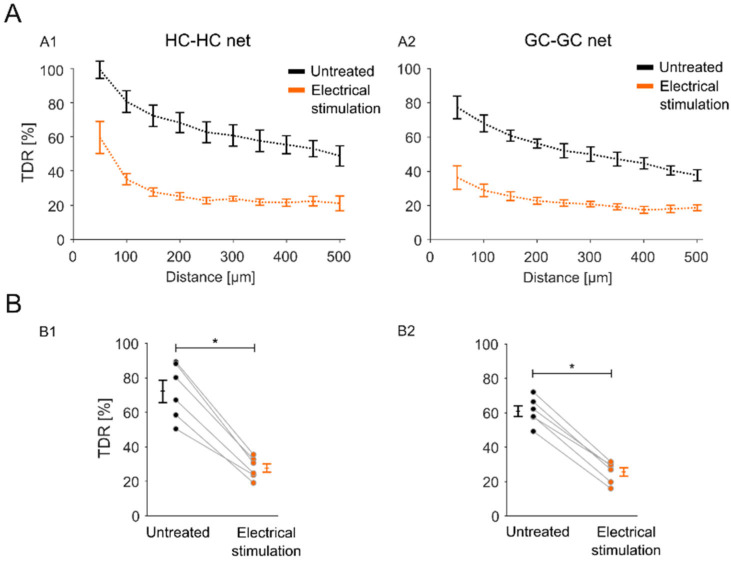
Spatial effect of electrical stimulation on retinal GJ-mediated tracer diffusion. (**A**) Stimulation electrode correlated with diffusion of the GJ tracer. Presentation of TDRs through the retinal GJ-net of (**A1**) HCs (HC-HC net) and (**A2**) GCs (GC-GC net) for the stimulation-treated retinal half (1 Hz, 1 V, and 1 h stimulation) and the untreated retinal half (*n* = 6 retinas; see Figure 6(C3) for electrode position). Therefore, each retina was imaged from the outer retina and the GC-layer side and the TDR was obtained from the corresponding layer image using the ROIs, as shown in Figure 4 (yellow squares). The X-axis represents the distance from the razor-cut site. Error bars indicate the mean ± SEM. (**B**) Impact of electrode location. Statistical evaluation of TDRs at 150 µm distance mark (from **A**), comparing the (**B1**) HC-HC net and (**B2**) GC-GC net of the stimulation-treated and untreated retinal halves. A Wilcoxon signed rank test was conducted to estimate the statistical significance (*: *p* < 0.05). Error bars indicate the mean ± SEM.

To evaluate whether the stimulation effect was correlated with the physical size of the stimulation electrode, retinal explants were stimulated in an overlap configuration (see Section 4.3. Experimental Conditions), in which only half of the explant was covered by the stimulation electrode. In the representative overview (Figure 4A), three distinguishable retinal parts are apparent (Figure 4A, separation line): the half with no tracer diffusion (region of interest (ROI 1)), the untreated half with tracer diffusion throughout the retina (ROI 3), and an intermediate zone with sparse tracer loading (ROI 2). The effect was significant to the position of the stimulation electrode (Figure 5) and was observed for the HC-HC net (Figure 5(A1,B1); TDR stimulated: 27.78 ± 2.55%; TDR untreated: 72.31 ± 6.59%) and the GC-GC net (Figure 5(A2,B2); TDR stimulated: 25.54 ± 54%; TDR untreated: 60.88 ± 3.24%).

In conclusion, the stimulation-induced reduction in retinal GJ permeability is a local effect and is related to the physical size and position of the stimulating electrodes.

## 3. Discussion

In the outer and inner plexiform layers, retinal cells communicate laterally via GJs [12,13,14]. Also known as electrical synapses, GJs form extraordinary multifunctional and tunable joints, gating ions (charge, [22,29,30,31]), and small molecules [26,27,28]; overall, they play a crucial role in health and disease. In a healthy retina, changes in the extent of the HC coupling allow retinal signal processing to switch modes from high to low light conditions [16]. In the inner retina, GJ-nets promote motion detection and synchronized GC activity. Furthermore, such tunable synapses are crucial for the denoising function of the early visual system and, overall, for shaping the visual signal at the originating level in the retina. The coupling of GJs also plays a significant role in the diagnostics of visual function, such as by electroretinogram [32]. GJ coupling shapes the electroretinogram’s oscillatory potentials [32], which are indicators of retinal diseases, including elevated intraocular pressure [33,34], different types of retinal dystrophy and degeneration, and early diagnosis of diabetic retinopathy [35,36,37,38].

Blinding diseases, such as retinitis pigmentosa, lead to the remodeling of the retinal tissue [17,19] after photoreceptor degeneration, and over a long period, a wild sprouting of retinal cells (e.g., HC [18]) can be observed to destroy natural synaptic structures, leading eventually to the rearrangement of retinal layers and connectivity (e.g., ectopic synapse [39]). Hence, for the development of an appropriate channel-targeted therapeutic strategy, such as curing blinding disease [2] or even restoring sight [5,6,7,8], it is crucial to understand the role and regulatory mechanism of the involved players.

Hence, we established an advanced GJ-tracer method inspired by previous works [25,26] that proved not only the existence of three GJ-nets in *rd1* retinas but also their functionality, and this allowed us to investigate the impact of sustained stimulation on the GJ’s permeability. Moreover, the versatile method will allow future studies to trace functional photoreceptor–photoreceptor coupling after transplantation [40]. Electrical stimulation has potential as a noninvasive and nonchemical treatment for GJ-related cortical neuronal diseases, such as epileptic seizures [41,42,43].

### 3.1. Lateral Signal Transduction in Degenerated rd1 Mouse Retinas

The common histological immunofluorescence labeling of GJs can prove only their presence but not their functionality or their cell coupling capability. In degenerated *rd1* retinas, the presence of GJs with AII ACs (Cx36) has been demonstrated in histological immunofluorescence preparations [44]. The anatomical data of HC suggest that the HC-HC GJ coupling [45] may be altered or even lost due to strong neuronal sprouting [17,18]. However, functional studies using GJ blockers have indicated some remaining functionality of the GJ-coupled networks, without identification of specific cell type or the span radius of the GJ cell coupling [20,21,22,23].

Hence, to resolve this seeming controversy, we established an advanced cell type–independent GJ-net tracking method using the tracer neurobiotin, which can permeate *only* GJs, and this method revealed vast GJ-nets in three different layers of the *rd1* retina. The identification of the cell network type was determined by identifying the retinal layers by their unique characteristics: the degenerated photoreceptor-less *rd1* outer retina with easily visual HCs [46,47,48] and occasionally dotted with pigment epithelium cells, and the GC layer with long GC axon fibers [49,50]. Between the HC-HC and GC-GC layers, we found a not-so-pronounced tracer-labeled layer, which we considered to be an AC-AC net since it was sufficiently spatially separated [51]. Additional cell identification criteria were the shape and the size of the labeled cells. The tracer-labeled cells matched their histological equivalents [46,47,48,49,50,51]. Although we identified the GJ-nets precisely, our calculated cell density was, on average, lower than that of previous studies using cell-specific immunofluorescence cell labeling (HC [46,47], AC [13,52], and GC [53,54]). The different numbers in our study might have resulted from the permeability state of the GJs and their functional coupling.

These findings prove that GJs are present and coupled, allowing intercellular connectivity in photoreceptor-degenerated *rd1* mouse retinas. To investigate their functionality further, we used the GJ blocker CBX. Inhibited tracer diffusion through the GJ-nets indicated functional GJs in the *rd1* retinas for all three GJ-nets.

We found high GJ permeability through all the retinal GJ-nets in the untreated blind retinal explants. We explain this as follows: in a healthy retina, the permeability of GJs, particularly HC-HC, is highest in darkness (GJ wide open) and is modulated by light-induced neuronal activity [15]. Hence, with the loss of light-driven activity, the extent of GJ coupling in photoreceptor-degenerated retinas may be comparable to that of a healthy dark-adapted retina [28,31]. The CBX-mediated elimination of GJ-nets confirmed the remaining functionality of GJs. It should be noted that we found that the GJ-nets were functional, but signs of disease were present in previous studies, generating aberrant cellular network activity in the photoreceptor-degenerated retinas [20,21,55,56], which could be abolished by targeting GJ-nets using blockers [22,28,55].

Overall, for the first time, our advanced GJ tracer method unequivocally proved in this study that photoreceptor-degenerated *rd1* mouse retinas (day 21–26) form GJ-nets of HC-HC, AC-AC, and GC-GC and that the GJ coupling among the cell networks is vast and functional.

### 3.2. Electrical Modulation of Retinal GJ-Nets

Sustained electrical stimulation diminished tracer diffusion through the GJ-nets, just as its chemical counterpart, CBX, proved that electrical stimulation can modulate the permeability of GJ-nets in *rd1* retinas. This effect has previously been reported for GJ coupling [57] and has been observed in the HC of healthy retinas [29,58,59,60].

In our approach, we assessed the voltage- and time-dependent effects. An optimal dose response was identified as the retinal stimulation of 1 V for 1 h at 1 Hz (safe and effective [31,61]), which abolished GJ-net coupling in all three layers. A lower voltage (0.5 V) for the same duration (1 h) or 1 V for a shorter period (30 min) did not show any significant effect on GJ-net permeability.

In a spatial context, the GC layer seems more susceptible to the reduction in GJ permeability induced by sustained stimulation than the outer retina (HC-HC). Nevertheless, the impact of the stimulation-induced reduction in GJ permeability was related to the stimulation electrode position (Figure 4).

Regarding the question of whether the modulatory effect of sustained stimulation on GJ-net permeability is direct or indirect, it is dependent on the cell type and cell layer. GJ permeability is not only regulated by voltage directly [29,58,59,60] (extracellularly, e.g., by means of an electrical field), but also intracellularly by calcium ions (calcium-calmodulin complex [62]). In the context of electrical stimulation, by targeting voltage-gated channels (VGCs), calcium enters retinal ACs and GCs via sodium- or calcium-dependent action potentials involving voltage-gated sodium channels (VGNaCs) and voltage-gated calcium channels (VGCCs), respectively [61,63]. HCs also express VGC (VGNaC and VGCC [63]); however, in vivo, they do not generate sodium- or calcium-dependent action potentials; nevertheless, it is conceivable that upon retinal electrical stimulation calcium ions may also enter HCs via their VGC. To this end, whether the electrical stimulation-evoked calcium transients affect the GJ coupling remains controversial [62]. On the one hand, it is reported that a cellular depletion of calcium leads to the closing of Cx [64]; on the other hand, a substantial increase in cellular calcium leads to the closing of Cx [65]. It is to be noted that these aforementioned findings were established for static calcium levels. In our experiments, however, oscillatory 1 Hz fluctuations of intracellular calcium were induced electrically (1 Hz stimulation frequency [31,61]). Calcium waves mediated by GJs have been observed in healthy GJ networks [66] as well as in diseased retinal tissue [22]. A difference between the artificially induced and the natural waves may lie in the higher amount of intruding calcium during sustained electrical stimulation, which in turn could affect the GJ coupling—however, this relation was not revealed in the presented study. Regarding the indirect modulation of GJs by neuromodulators, the HC-HC net is regulated by dopamine [67,68], while for the AC-AC and GC-GC networks a secondary modulator is unknown. An effect of the inhibitory neurotransmitters gamma-aminobutyric acid (GABA) or glycine can also be excluded since they are not known to block GJs [22], and neither the HCs nor the ACs express receptors for inhibitory neurotransmitters [69].

This study demonstrated that extracellular *sustained* stimulation could modulate GJ permeability in retinal explants. This approach was as effective as its chemical counterpart, the GJ blocker CBX. In contrast with the global systemic effect of CBX, the stimulation’s inhibitory effect was local to the stimulation electrode.

### 3.3. Implications of Electrical Stimulation-Mediated Modulation of GJ-Nets for the Development of Strategies for Electrical Implants

The permeability of GJs to small molecules was demonstrated in this work by the vast diffusion of the GJ tracer through the three retinal GJ-nets. This cell coupling over a large retinal area also has substantial implications for the applications that interface neurons with electrical stimulation. Thus, the permeability of GJs to ions also allows the lateral spread of electrical voltage through GJ-coupled neighboring cells [29,30,70], which allows even synchronous calcium oscillations over a large network space (HC-HC [22], GC-CG [21], and AC-AC [56]). A specific example of the lateral spread of electrically induced retinal activity was presented by Haq et al. [31]. The spread was reduced to the stimulation electrode’s vicinity by applying the GJ blocker CBX [22,55].

As sustained stimulation blocked tracer diffusion through the retinal GJ-nets, as occurs with its chemical counterpart, CBX, and since CBX abolished the lateral spread of GJ-mediated activity, it is very likely that the application of electrical stimulation in the longer term would eventually cause the reshaping of the retinal signaling pathway (by direct electrical modulation and/or by secondary transmitter modulation). This effect may be beneficial in reducing aberrant cellular noise in neuroretinal networks mediated by a degenerative disease, such as photopsia [20,21,28,55,56,71,72], and thereby increasing the signal-to-noise ratio.

Furthermore, it is worth noting that the adverse effects of stimulation-induced GJ blockage due to the prevention of small molecules (crucial for retinal vital processes) from diffusing were not investigated within this work. Nevertheless, in previous studies, a knockout of photoreceptor–photoreceptor GJ (Cx36) did not lead to cell death [73], while a knockout of GJ connexin in the inner retina led to cell deterioration [74]. Nonetheless, adequate cyclic stimulation (controlling certain permeability), including sufficient pauses, could solve the problem, since the closure and opening of GJs is a natural process.

Overall, these effects should be considered carefully in medical approaches in which sustained electrical stimulation is delivered to excitable neuronal tissue facilitating GJ-nets, such as through retinal implants [5,6], transcorneal electrical stimulation [75,76], cochlear implants [77,78], cortical implants for vision [7], and cortical seizures [41,43].

## 4. Materials and Methods

### 4.1. Animals

This study was conducted in the *rd1* (C3H Pde6b*^rd1/rd1^*) mouse model for retinal degeneration [17,24] (blind; age: 21–26 days; *n* = 13). The animals were housed under standard light conditions with free access to water and food and were used regardless of gender. They were sacrificed in a carbon dioxide atmosphere followed by cervical dislocation, and both eyes were carefully enucleated.

### 4.2. Retinal Tissue Preparation

The retinas were isolated in artificial cerebrospinal fluid (ACSF) containing (in mM) 125 NaCl, 26 NaHCO_3_, 2.5 KCl, 2 CaCl_2_, 1 MgCl_2_, 1.25 NaH_2_PO_4_, and 20 glucose (all the chemicals were purchased from Sigma-Aldrich, Darmstadt, Germany). The pH was kept at 7.4 by carbogen perfusion (95% CO_2_/5% O_2_). The retinal explants were cut in half to reduce animal numbers before starting the experiment. The retinal explants were placed on filter paper (nitrocellulose membrane, 0.8 µm pores, Merck Millipore, Tullagreen Carrigtwohill, Ireland), GC side down.

### 4.3. Experimental Conditions

**Figure 6:**
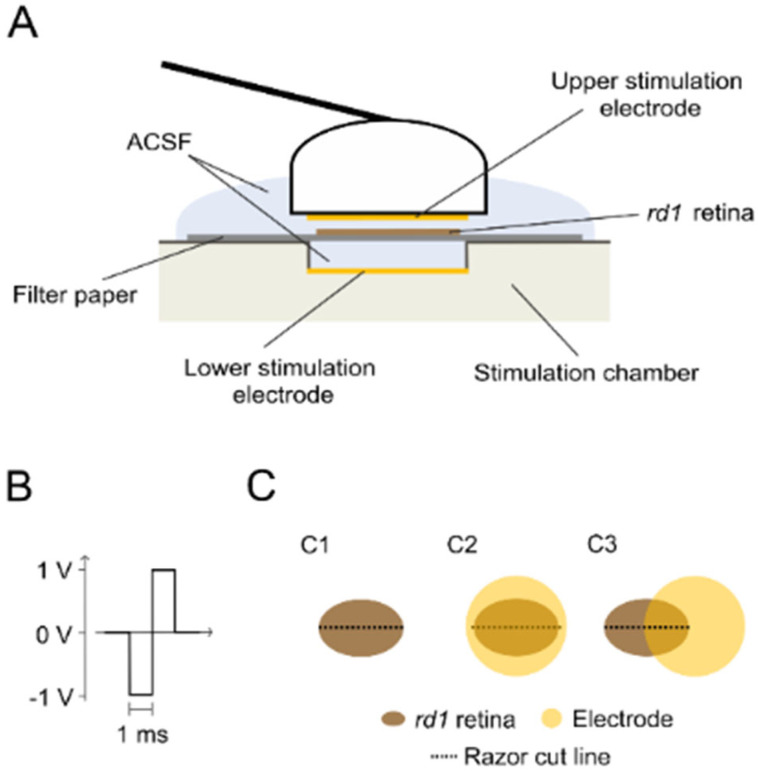
Sketch of the experimental setup. (**A**) Application of electrical stimulation. Retinal explants of the blind *rd1* mice were placed on filter paper (GC side down) and positioned between the two stimulation electrodes (upper and lower electrode: 5 mm diameter; electrode spacing to filter paper: 1 mm for the upper electrode and 3 mm for the lower electrode; used medium: artificial cerebrospinal fluid (ACSF)). The upper electrode shaft was driven by a micromanipulator. (**B**) Stimulation parameters. For retinal stimulation, a voltage-balanced biphasic pulse (cathodic phase first; 1 ms per phase) was used. Sustained stimulation was applied at a frequency of 1 Hz and at 0.5 V or 1 V for different durations (0.5 h, 1 h, or 2 h). (**C**) Electrode location. Experiments were carried out (**C1**) without stimulation treatment of the *rd1* retinal explant or with stimulation treatment (**C2**) with the electrode fully covering the retinal explant or (**C3**) with the electrode covering half of the retinal explant. The razor cut through the retinal explant (indicated by a black dashed line) was performed after treatment and before tracer loading.

The untreated (control condition) retinal explants rested on filter paper under a carbogen atmosphere for 1 h. To investigate retinal GJ coupling, the GJ inhibitor CBX (150 μM solved in ACSF, (3β,20β)-3-(3-Carboxy-1-oxopropoxy)-11-oxoolean-12-en-29-oic acid disodium, Sigma-Aldrich, Darmstadt, Germany) was bath applied to the retinal explants under a carbogen atmosphere for 1 h. Electrical stimulation of the retinal explants was applied using a custom-made setup (Figure 6A). The setup consisted of a base chamber with an integrated electrode (referred to as the lower electrode) and a single large electrode (both electrodes: diameter 5 mm; platinum, Nepa Gene, Ichikawa-City, Japan) mounted on a shaft (referred to as the upper electrode). The retina mounted on filter paper was placed on top of the lower electrode (3 mm distance to the lower electrode); then, the upper electrode, attached to a micromanipulator, was lowered toward the retina (1 mm distance to filter paper). A supply of fresh carbogen-perfused ACSF was provided throughout the stimulation procedure. The stimulation protocol was defined with MC Stimulus II software (v 3.4.4) and generated by a stimulus generator (STG 2008, both hardware and software Multi-Channel Systems, Reutlingen, Germany). An oscilloscope (TDS2014C, Tektronix, Beaverton, OR, USA) was connected to monitor the stimulation. The stimulation pulse type was biphasic and voltage-balanced (cathodic first, 1 ms/phase, Figure 6B). The sustained stimulation was delivered at 1 Hz, with varied strengths (0.5 V or 1 V) and duration (0.5 h, 1 h, or 2 h). Stimulation was applied in two different electrode configurations: covering the retinal tissue entirely or overlapping partly (Figure 6C). All the experiments were performed at room temperature.

### 4.4. Retinal Tracer Loading

To assess the retinal GJ-nets, we established the method of tracer loading (adapted from Choi et al. [25]). The retinal explants were cut with a razor blade to open the cells for the loading of the tracer neurobiotin (Vector Laboratories, Newark, CA, USA), which can permeate through GJ *only*, not the cell membrane. The retinal explants were exposed to 200 µL of tracer solution (0.5% neurobiotin dissolved in ACSF) for 5 min and then washed twice for 20 min under a carbogen atmosphere. Subsequently, the retinal explants were fixed with 4% paraformaldehyde (PFA, Morphisto GmbH, Offenbach am Main, Germany) for 1 h and then washed 3 times for 20 min in phosphate-buffered saline (PBS, 0.1 M, custom-made, chemicals from Sigma-Aldrich, Darmstadt, Germany). The fixated retinal explants were incubated overnight at 4 °C with streptavidin-conjugated Alexa 488 (pure, Molecular Probes Inc., Thermo Fisher Scientific, Darmstadt, Germany) for fluorescent tagging of the tracer neurobiotin. Next, the retinal explants were washed twice for 30 min in PBS and mounted with Fluoromount-G mounting medium (Invitrogen, Thermo Fisher Scientific, Darmstadt, Germany) on object slices (76 × 26 mm, pre-cleaned, R. Langenbrinck GmbH, Emmendingen, Germany).

### 4.5. Fluorescence Imaging

The fluorescence microscope imaging system Axio Imager Z1 (Carl Zeiss AG, Oberkochen, Germany; filter set detecting Alexa 488 (λ_exc_ = 488 nm, λ_em_ = 509 nm)) was used to image the retinal fluorescence. Large field images of the retinal explants (mosaic of single images) were obtained using a plan apochromat 5×/0.16 objective (pixel size: 1.290 × 1.290, bit depth: 12 bit). Both the outer retina and the GC side were imaged. Additionally, high-resolution image stacks (*z*-axis throughout retinal depth, 5 µm steps) were obtained utilizing a plan apochromat 20×/0.8 M27 objective to assess cells in different retinal layers.

### 4.6. Data Analysis

Cell counting was performed using ZEISS ZEN software (v 3.9, Carl Zeiss AG, Oberkochen, Germany) in 200 µm^2^ ROIs, with a 150 µm distance to the razor-cut site in cell layer images extracted from the image stacks. We estimated the tracer diffusion through the retinal networks to quantify GJ permeability. To accomplish this, the ROIs were set vertically to the razor-cut site in the large field images, and fluorescence value profiles were extracted (mean over the ROI width of 200 µm; ROI depth: 500 µm into the tissue; 1.3 µm steps; 3 ROIs in each retinal explant) using ZEISS ZEN software. The ROIs were set behind the first line of dye-filled cells at the razor cut, as those were overexposed in the images. The ROI profiles were extracted from ZEISS ZEN and processed in MATLAB (MathWorks, R2020a, Natick, MA, USA). The TDR was computed by normalizing the extracted profiles against the first value from the cut site and was presented as a smoothed (Savitzky–Golay) profile per retina (grand mean of 3 ROIs in each retinal explant). A Wilcoxon signed rank test was conducted for statistical analysis of the data obtained from the retinal explants treated in the overlapping-electrode configuration. For all other conditions, a one-way analysis of variance (ANOVA) was conducted, followed by Dunnett’s test for multiple comparisons.

## 5. Conclusions

The GJs of photoreceptor-degenerated *rd1* mouse retinas are functional and form coupled networks of HC-HC, AC-AC, and GC-GC. Their coupling allows the transition of small molecules (as demonstrated by the GJ tracer) and ions [22]; this transition was abolished by extracellular electrical stimulation in a similar manner to that of its chemical counterpart.

When designing medical applications for cases where potent drugs are unavailable or a replacement is required due to side effects, electrical stimulation has great potential as a noninvasive and nonchemical replacement. It could potentially control the wide spread of malign small messenger molecules through GJ-coupled tissue [79] and reduce the lateral spread of unwanted aberrant cellular noise, such as that caused by photopsia [20,21,55,56] or cortical seizures [41,43].

Moreover, the impact of sustained long-term stimulation on the shaping of the neuronal signaling pathways is key for strategies developing stimulation paradigms [80] for the rehabilitation of sensory inputs by electrical stimulation, such as retinal, cochlear, and cortical implants. Furthermore, as an advanced application, the tracer method would allow the study of the challenging functionality of photoreceptor–photoreceptor coupling after transplantation [8].

## Data Availability

The data presented in this study are available in the article.

## Supplementary Materials

The supporting information can be downloaded at: https://www.mdpi.com/article/10.3390/ijms25031616/s1.

## Abbreviations

The following abbreviations are used in this manuscript.

GJs	gap junctions
GJ-net	gap junction-coupled network
*rd1*	retinal degeneration 1
dPhr	degenerated photoreceptor
HC	horizontal cell
BC	bipolar cell
AC	amacrine cell
GC	ganglion cell
ACSF	artificial cerebrospinal fluid
PBS	phosphate-buffered saline
CBX	carbenoxolone
M	molar
mM	millimolar
µm	micrometer
mm	millimeter
µl	microliter
h	hour
min	minute
V	volt
ROI	region of interest
TDR	tracer diffusion rate
VGC	voltage gated channel
VGNaC	voltage gated sodium channel
VGCC	voltage gated calcium channel
ANOVA	analysis of variance
SEM	standard error of the mean
STD	standard deviation
