# Supplementary material for: Sustained Extracellular Electrical Stimulation Modulates the Permeability of Gap Junctions in rd1 Mouse Retina with Photoreceptor Degeneration
Source: Int J Mol Sci. 2024 Jan 28;25(3):1616. doi: 10.3390/ijms25031616 (PMC10855676; doi:10.3390/ijms25031616)
Supplement: Supplementary file 1 [file ijms-25-01616-s001.zip › ijms-2827233-supplementary.pdf]

Sustained Extracellular Electrical Stimulation Modulates the Permeability of Gap Junctions in *rd1* Mouse Retina with Photoreceptor Degeneration

Sophie Stürmer, Sylvia Bolz, Eberhart Zrenner, Marius Ueffing and Wadood Haq *

Institute for Ophthalmic Research, University of Tuebingen, 72076 Tuebingen, Germany; sophie.stuermer@uni-tuebingen.de (S.S.); sylvia.bolz@uni-tuebingen.de (S.B.); ez@uni-tuebingen.de (E.Z.); marius.ueffing@uni-tuebingen.de (M.U.)
* Correspondence: wadood.haq@uni-tuebingen.de (W.H.)

Table S1. P-values of statistical data presented in Figure 4. Statistical evaluation of tracer diffusion rate (TDR, Figure 4A) at 150 µm mark for (Figure 4B1) HC-HC net and (Figure 4B2) GC-GC net assessed under different treatment conditions. One-way ANOVA was applied to estimate the statistical significance, followed by a Dunnett's test for multiple comparisons. Significance levels: not significant (n.s.), *: $p < 0.05$, **: $p < 0.01$ and ***: $p < 0.001$.

**A) Statistical analysis of tracer diffusion rates in the outer retina**						
	**Control**	**0.5 V, 1 h**	**1 V, 0.5 h**	**1 V, 1 h**	**1 V, 2 h**	**CBX**
**Control**		0.74 (n.s.)	1.00 (n.s.)	5.26E-11 (***)	1.15E-08 (***)	3.41E-12 (***)
**0.5 V, 1 h**	0.74 (n.s.)		0.98 (n.s.)	3.52E-08 (***)	4.68E-06 (***)	1.05E-09 (***)
**1 V, 0.5 h**	1.00 (n.s.)	0.98 (n.s.)		4.62E-09 (***)	4.13E-07 (***)	1.89E-10 (***)
**1 V, 1 h**	5.26E-11 (***)	3.52E-08 (***)	4.62E-09 (***)		1.00 (n.s.)	0.68 (n.s.)
**1 V, 2 h**	1.15E-08 (***)	4.68E-06 (***)	4.13E-07 (***)	1.00 (n.s.)		0.20 (n.s.)
**CBX**	3.41E-12 (***)	1.05E-09 (***)	1.89E-10 (***)	0.68 (n.s.)	0.20 (n.s.)	
**B) Statistical analysis of tracer diffusion rates in the ganglion cell layer**						
	**Control**	**0.5 V, 1 h**	**1 V, 0.5 h**	**1 V, 1 h**	**1 V, 2 h**	**CBX**
**Control**		0.94 (n.s.)	0.32 (n.s.)	8.75E-07 (***)	9.14E-06 (***)	3.60E-07 (***)
**0.5 V, 1 h**	0.94 (n.s.)		1.00 (n.s.)	9.06E-05 (***)	5.26E-04 (***)	2.52E-05 (***)
**1 V, 0.5 h**	0.32 (n.s.)	1.00 (n.s.)		9.72E-03 (**)	2.40E-02 (*)	2.11E-03 (**)
**1 V, 1 h**	8.75E-07 (***)	9.06E-05 (***)	9.72E-03 (**)		1.00 (n.s.)	1.00 (n.s.)
**1 V, 2 h**	9.14E-06 (***)	5.26E-04 (***)	2.40E-02 (*)	1.00 (n.s.)		1.00 (n.s.)
**CBX**	3.60E-07 (***)	2.52E-05 (***)	2.11E-03 (**)	1.00 (n.s.)	1.00 (n.s.)	
